# Association of Nasopharyngeal and Serum Glutathione Metabolism with Bronchiolitis Severity and Asthma Risk: A Prospective Multicenter Cohort Study

**DOI:** 10.3390/metabo12080674

**Published:** 2022-07-22

**Authors:** Michihito Kyo, Zhaozhong Zhu, Makiko Nanishi, Ryohei Shibata, Tadao Ooka, Robert J. Freishtat, Jonathan M. Mansbach, Carlos A. Camargo, Kohei Hasegawa

**Affiliations:** 1 Department of Emergency Medicine, Massachusetts General Hospital, Harvard Medical School, Boston, MA 02114-1101, USA; zzhu5@mgh.harvard.edu (Z.Z.); mnanishi@mgh.harvard.edu (M.N.); rshibata@mgh.harvard.edu (R.S.); tooka@mgh.harvard.edu (T.O.); ccamargo@partners.org (C.A.C.J.); khasegawa@mgh.harvard.edu (K.H.); 2 Department of Health Science, University of Yamanashi, Chuo, Yamanashi 409-3898, Japan; 3 Department of Genomics and Precision Medicine, George Washington University, Washington, DC 20052, USA; rfreishtat@childrensnational.org; 4 Division of Emergency Medicine, Children’s National Hospital, Washington, DC 20010, USA; 5 Department of Pediatrics, Boston Children’s Hospital, Harvard Medical School, Boston, MA 02115, USA; jonathan.mansbach@childrens.harvard.edu

**Keywords:** asthma, bronchiolitis, glutathione, infant, metabolome, oxidative stress, severity

## Abstract

Infants hospitalized for bronchiolitis are at high risk for asthma. Glutathione-related metabolites may antagonize oxidative stress, which induces airway injuries in respiratory infection and subsequent airway remodeling. However, little is known about the relationship of glutathione-related metabolites with bronchiolitis severity and the risk of asthma. In a multicenter prospective observational cohort study of infants hospitalized for bronchiolitis, we measured nasopharyngeal and serum glutathione-related metabolites by using liquid chromatography–tandem mass spectrometry. We then examined their association with bronchiolitis severity (defined by positive pressure ventilation (PPV) use). We also identified severity-related glutathione-related metabolite signatures and examined their association with asthma at age 6 years. In 1013 infants, we identified 12 nasopharyngeal and 10 serum glutathione-related metabolites. In the multivariable models, lower relative abundances of seven metabolites, e.g., substrates of glutathione, including cysteine (adjOR 0.21, 95%CI 0.06–0.76), glycine (adjOR 0.25, 95%CI 0.07–0.85), and glutamate (adjOR 0.25, 95%CI 0.07–0.88), were significantly associated with PPV use (all FDR < 0.05). These associations were consistent with serum glutathione-related metabolites. The nasopharyngeal glutathione-related metabolite signature was also associated with a significantly higher risk of asthma (adjOR 0.90, 95%CI 0.82–0.99, *p* = 0.04). In infants hospitalized for bronchiolitis, glutathione-related metabolites were associated with bronchiolitis severity and asthma risk.

## 1. Introduction

Bronchiolitis is the leading cause of hospitalizations for U.S. infants, accounting for 110,000 hospitalizations each year [1]. Approximately 7% of infants hospitalized for bronchiolitis undergo positive pressure ventilation (PPV) [2]. Its chronic morbidity burden is also notable; 30%–40% of these hospitalized infants subsequently develop childhood asthma [3,4,5,6,7,8,9,10,11,12,13]. The literature has also documented that higher severity of bronchiolitis is related to a higher risk of asthma [14,15]. However, the mechanisms underlying these two conditions remain uncertain.

Of the potential pathobiological pathways, glutathione metabolism plays an antioxidative role in removing intracellular oxidants [16] that induce airway injuries in acute respiratory infection [17] and subsequently airway remodeling [18]. However, there have only been a few studies that have evaluated the role of glutathione metabolism in bronchiolitis. An in vitro experiment has reported that the ratio of glutathione to oxidized glutathione (glutathione disulfide) decreased in small airway epithelial cells with respiratory syncytial virus (RSV) infection [19]. Additionally, a single-center case–control study [20] found higher serum glutathione disulfide levels in infants with RSV bronchiolitis who received supplemental oxygen than those who did not. Despite the apparent clinical and research importance, the role of glutathione-related metabolites in the pathobiology of infant bronchiolitis and asthma development remains unclear.

To address this knowledge gap, we aimed to investigate the relationship of glutathione-related metabolites with bronchiolitis severity (i.e., use of PPV [21] and intensive care [22]) and childhood asthma development by examining both nasopharyngeal and serum metabolome data from a multicenter observational cohort. A better understanding of the role of glutathione may inform potential strategies to develop an effective treatment for bronchiolitis and thereby prevent asthma in this high-risk population.

## 2. Results

### 2.1. Patient Characteristics

Data from a multicenter prospective observational cohort study of infants hospitalized for bronchiolitis—the 35th Multicenter Airway Research Collaboration (MARC-35) study [15,21,23,24,25]—were analyzed. This study enrolled infants (age < 1 year) who were hospitalized with bronchiolitis at 17 sites across 14 U.S. states. The current analysis investigated 1013 infants who underwent nasopharyngeal metabolome profiling and a subset of 140 infants who underwent serum metabolome profiling [23]. The clinical outcomes of interest were acute severity of bronchiolitis and development of asthma by age 6 years.

Of 1013 infants who underwent nasopharyngeal metabolome profiling, the median age was 3 months (IQR 2–6 months), 40% were female, and 81% had RSV infection (Table 1). Additionally, 5% underwent PPV (defined as the use of continuous positive airway pressure ventilation and/or mechanical ventilation [21])—the primary clinical outcome—and 16% received intensive care (defined as an intensive care unit admission and/or PPV use [22])—the secondary outcome—during the hospitalization for bronchiolitis. In addition, 32% developed asthma (defined as a commonly used epidemiologic definition [26,27,28]) by the age of 6 years. Of these patients, 140 infants also underwent serum metabolome profiling.

### 2.2. Associations of Glutathione-Related Metabolites with Severity Outcomes

**Figure 1:**
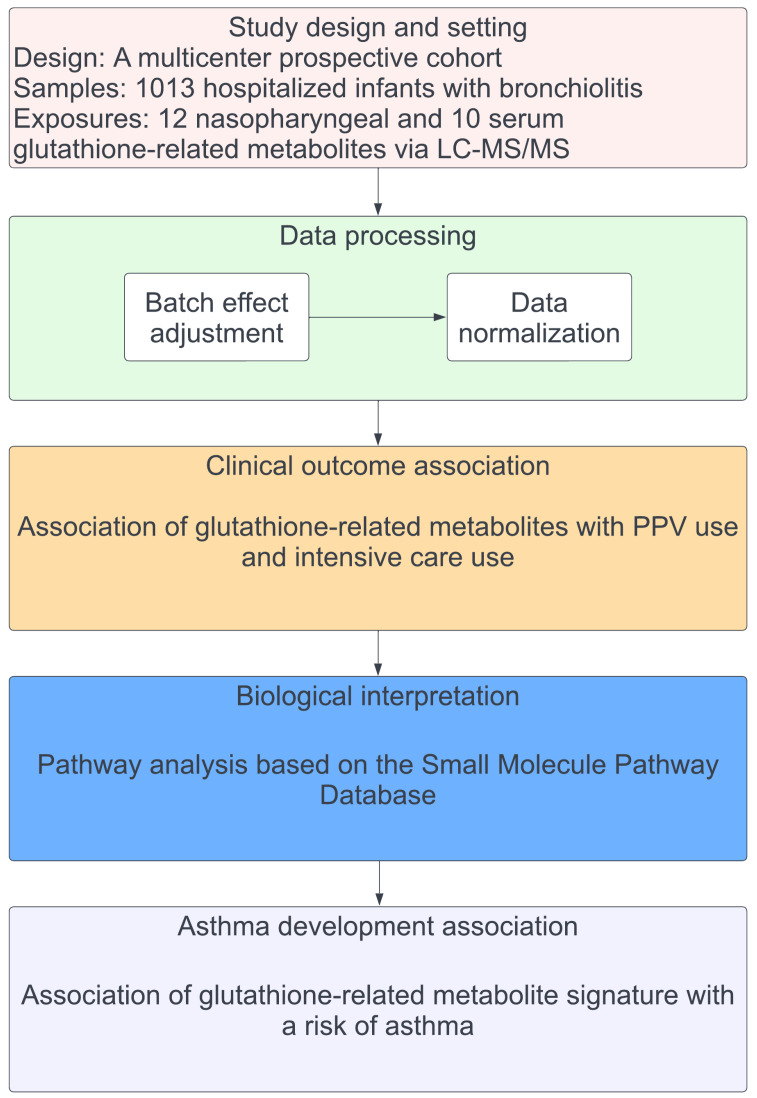
**Analytic workflow.** The analytic cohort consisted of 1013 infants hospitalized for bronchiolitis in a multicenter prospective cohort study—MARC-35. In this cohort, 12 nasopharyngeal airway and 10 serum glutathione-related metabolites were identified by using liquid chromatography–tandem mass spectrometry (LC-MS/MS). The glutathione-related metabolites data were adjusted for the potential batch effect and normalized. The association of the glutathione-related metabolites with the risk of PPV use and intensive care use was estimated. To examine the biological function of the measured metabolites, metabolite set enrichment analysis was conducted. The nasopharyngeal and serum glutathione-related metabolite signatures for PPV use were estimated. Then, the association of the signature with the risk of developing asthma was estimated. Abbreviations: LC-MS/MS, liquid chromatography–tandem mass spectrometry; PPV, positive pressure ventilation.

The nasopharyngeal and serum metabolome profiling was conducted using liquid chromatography–tandem mass spectrometry (LC-MS/MS). The analytic workflow is summarized in Figure 1. From the metabolome data, 12 nasopharyngeal and 10 serum glutathione-related metabolites (Figure S1) were identified, referring to the Kyoto Encyclopedia of Genes and Genomes (KEGG) pathway database [29]. The glutathione-related metabolites data were adjusted for the potential batch effect and normalized by total sum scaling [30] for nasopharyngeal samples and log transformation [23] for serum samples.

**Figure 2:**
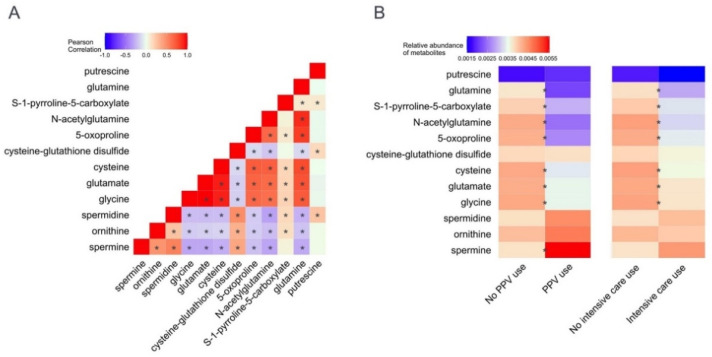
**Correlations between nasopharyngeal glutathione-related metabolites and associations of glutathione-related metabolites with severity outcomes in infants hospitalized for bronchiolitis.** (**A**). The heatmap shows the correlation between 12 measured nasopharyngeal glutathione-related metabolites. Glutathione substrates (i.e., cysteine, glycine, and glutamate) were positively correlated with each other. In contrast, these metabolites were negatively correlated with oxidized glutathione state (cysteine-glutathione disulfide). * *p*-value < 0.05 that is estimated by Pearson correlation coefficient. (**B**). The heatmap shows the association of nasopharyngeal glutathione-related metabolites with each of the two clinical outcomes. The relative abundance of glutathione substrates (i.e., cysteine, glycine, and glutamate) was lower in the PPV use group and the intensive care use group compared to their reference groups, suggesting their depletion. In contrast, the relative abundance of metabolites that bind glutathione (e.g., spermine) was higher in the PPV use group. The association of each metabolite with each outcome is shown in Figure 3. * FDR < 0.05 estimated by unadjusted logistic regression models. Abbreviations: FDR, false discovery rate; PPV, positive pressure ventilation.

To examine the correlations within the nasopharyngeal and serum glutathione-related metabolites, Pearson correlation coefficients were computed. Of the nasopharyngeal glutathione-related metabolites, cysteine, glycine, and glutamate (glutathione substrates) were significantly positively correlated with each other (all *p* < 0.05; Figure 2A). In contrast, these glutathione substrates were significantly negatively correlated with cysteine-glutathione disulfide (oxidized glutathione state) (all *p* < 0.05). Serum glutathione-related metabolites had similar patterns (Figure S2A).

**Figure 3:**
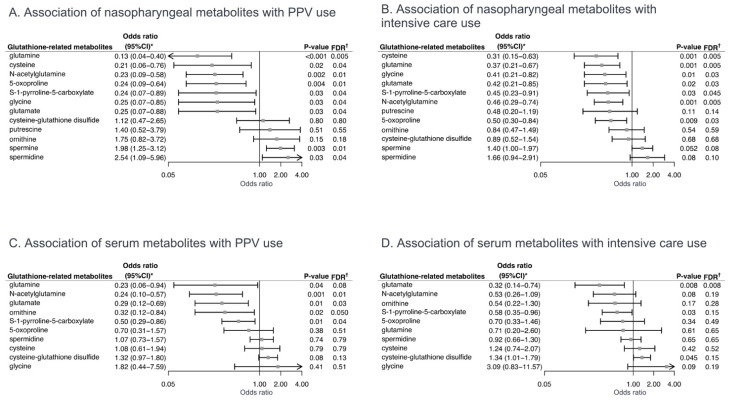
**Adjusted associations of nasopharyngeal and serum glutathione-related metabolites with severity outcomes in infants hospitalized for bronchiolitis.** (**A**). Multivariable-adjusted association of nasopharyngeal glutathione-related metabolites with PPV use. The ORs were estimated for a 1% change in the relative abundance of metabolites. (**B**). Multivariable-adjusted association of nasopharyngeal glutathione-related metabolites with intensive care use. The ORs were estimated for a 1% change in the relative abundance of metabolites. (**C**). Multivariable-adjusted association of serum glutathione-related metabolites with PPV use. The ORs were estimated in a 2-fold change in the abundance of metabolites. (**D**). Multivariable-adjusted association of serum glutathione-related metabolites with intensive care use. The ORs were estimated in a 2-fold change in the abundance of metabolites. Arrows indicate that the 95%CI of the odds ratio exceeds the lower or higher limit of the *x*-axis. * Estimated by fitting logistic regression model adjusting for potential confounders (age, sex, and RSV infection). † The Benjamini–Hochberg FDR method was used to account for multiple testing. Abbreviations: CI, confidence interval; FDR, false discovery rate; OR, odds ratio; PPV, positive pressure ventilation; RSV, respiratory syncytial virus.

**Figure 4:**
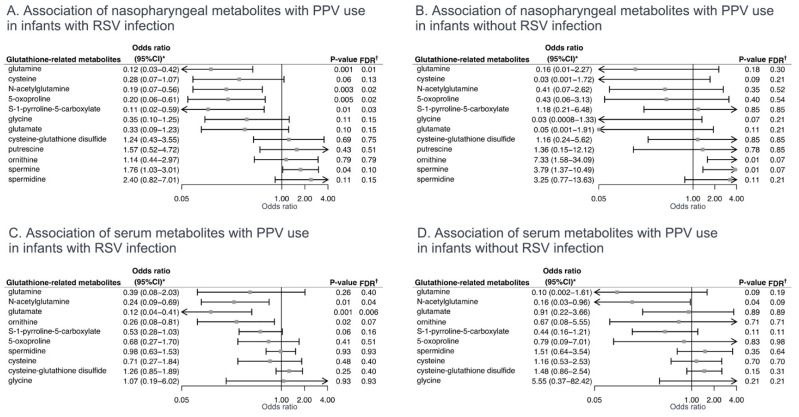
**Stratified analysis of associations of nasopharyngeal and serum glutathione-related metabolites with severity outcome in infants hospitalized for bronchiolitis, by respiratory syncytial virus infection.** (**A**). Multivariable-adjusted association of nasopharyngeal glutathione-related metabolites with PPV use in infants with RSV infection. The ORs were estimated for a 1% change in the relative abundance of metabolites. (**B**). Multivariable-adjusted association of nasopharyngeal glutathione-related metabolites with PPV use without RSV infection. The ORs were estimated for a 1% change in the relative abundance of metabolites. (**C**). Multivariable-adjusted association of serum glutathione-related metabolites with PPV use with RSV infection. The ORs were estimated in a 2-fold change in the abundance of metabolites. (**D**). Multivariable-adjusted association of serum glutathione-related metabolites with PPV use without RSV infection. The ORs were estimated in a 2-fold change in the abundance of metabolites. Arrows indicate that the 95%CI of the odds ratio exceeds the lower or higher limit of the *x*-axis. * Estimated by fitting logistic regression model adjusting for potential confounders (age and sex). † The Benjamini–Hochberg FDR method was used to account for multiple testing. Abbreviations: CI, confidence interval; FDR, false discovery rate; OR, odds ratio; PPV, positive pressure ventilation; RSV, respiratory syncytial virus.

To investigate the associations of nasopharyngeal and serum glutathione-related metabolites with the clinical outcomes of interest, unadjusted and multivariable logistic regression models were fit. Of the nasopharyngeal glutathione-related metabolites, the relative abundances of cysteine, glycine, and glutamate were significantly lower in both the PPV use and intensive care use groups, compared to the reference groups (all false discovery rates (FDRs) < 0.05; Figure 2B). In contrast, the relative abundance of spermine, which binds glutathione, was significantly higher in the PPV use group (FDR < 0.05). In the multivariable models, the lower relative abundances of seven nasopharyngeal metabolites, e.g., cysteine (adjOR 0.21, 95%CI 0.06–0.76), glycine (adjOR 0.25, 95%CI 0.07–0.85), and glutamate (adjOR 0.25, 95%CI 0.07–0.88), were significantly associated with PPV use (FDR < 0.05; Figure 3A). In the stratified analysis by RSV infection, the direction of the association was consistent with wider 95%CIs in the setting of the limited statistical power (Figure 4).

Of the serum glutathione-related metabolites, their association with bronchiolitis severity was similar but weaker. For example, the relative abundance of glutamate was non-significantly lower in the PPV use group (all FDR = 0.06; Figure S2B). In the multivariable models, the lower relative abundances of three metabolites, e.g., glutamate (adjOR 0.29, 95%CI 0.12–0.69, FDR = 0.03), were significantly associated with PPV use. In contrast, the higher relative abundance of cysteine-glutathione disulfide was non-significantly associated with the risk of PPV use (adjOR 1.32, 95%CI 0.97–1.80, FDR = 0.13; Figure 3C) and intensive care use (adjOR 1.34, 95%CI 1.01–1.79, FDR = 0.15; Figure 3D).

### 2.3. Metabolite Set Enrichment Analysis

**Figure 5:**
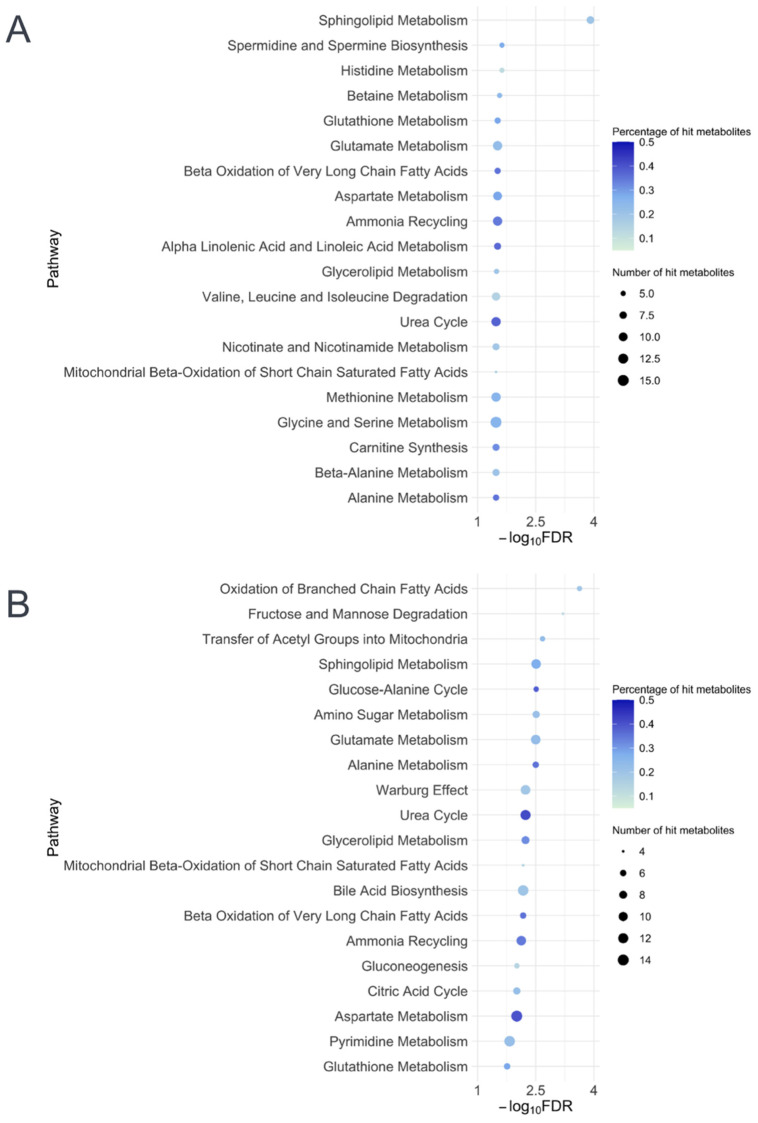
**Pathway analysis of nasopharyngeal and serum metabolites differentially enriched among infants who underwent positive pressure ventilation.** The shown metabolite pathways (based on the SMPDB) are the top 20 (**A**) nasopharyngeal and (**B**) serum pathways with the smallest FDRs that have the number of hits per pathway of >4 metabolites and the percentage of hits per total compound of >10%. The color of each dot represents the proportion of hit metabolites; the size of each dot represents the number of hit metabolites. The glutathione pathway was significantly differentially enriched by the positive pressure ventilation outcome in both nasopharyngeal and serum metabolome data (both FDR < 0.05). Abbreviations: FDR, false discovery rate; SMPDB, Small Molecule Pathway Database.

To examine whether the glutathione metabolism pathway is enriched in infants with higher bronchiolitis severity, metabolite set quantitative enrichment analyses [31] by using the Small Molecule Pathway Database [32] were performed. The metabolite set enrichment analyses demonstrated that the glutathione metabolism pathway was significantly associated with PPV use in both the nasopharyngeal and serum data (both FDR < 0.05; Figure 5). For example, glutathione metabolism was the fifth most significantly enriched pathway according to the nasopharyngeal metabolome data, which supports the clinical significance of glutathione-related metabolites in infant bronchiolitis.

### 2.4. Association of Severity-Related Glutathione Metabolite Signatures with Asthma

**Figure 6:**
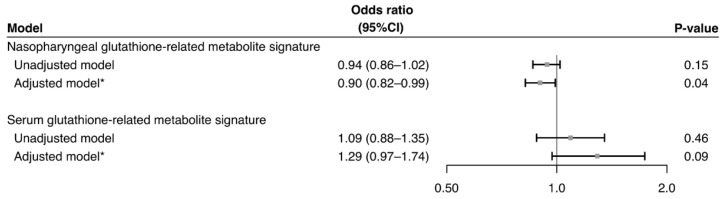
**Association of glutathione-related metabolite signatures with risk of developing asthma.** The nasopharyngeal and serum glutathione-related metabolite signatures for PPV use were estimated by constructing generalized additive models. Then, the association of the signature with the risk of developing asthma was estimated by fitting logistic regression models. The ORs were estimated in a change in the weighted sum of the glutathione-related metabolite coefficients. * Estimated by fitting logistic regression model adjusting for potential confounders (age, sex, race/ethnicity, prematurity, daycare use, cigarette smoke exposure at home, maternal smoking during pregnancy, parent history of eczema, RSV infection, RV infection, and any IgE sensitization). Abbreviations: CI, confidence interval; IgE, Immunoglobulin E; OR, odds ratio; PPV, positive pressure ventilation; RSV, respiratory syncytial virus; RV, rhinovirus.

Nasopharyngeal and serum PPV-related glutathione-related metabolite signatures were computed by using the generalized additive model. The predictive ability of the nasopharyngeal signature was an area under the receiver-operating-characteristics curve (AUC) of 0.82 (95%CI 0.76–0.88; Figure S3A); that of the serum signature was 0.88 (95%CI 0.81–0.95; Figure S3B). In the multivariable logistic regression model, the nasopharyngeal signature was negatively associated with the risk of asthma development (adjOR 0.90, 95%CI 0.82–0.99, *p*-value = 0.04; Figure 6).

## 3. Discussion

Based on the multicenter analysis of nasopharyngeal and serum metabolome data from a multicenter prospective observational cohort study of 1013 infants hospitalized for bronchiolitis, we identified nasopharyngeal and serum glutathione-related metabolites that were associated with bronchiolitis severity—defined by PPV use and intensive care use. In particular, the depletion of glutathione substrates (e.g., cysteine, glycine, glutamate) was associated with higher severity. In contrast, a higher relative abundance of oxidized glutathione state (cysteine-glutathione disulfide) was associated with non-significantly higher severity. Furthermore, we also found that the nasopharyngeal metabolite signature for acute severity was significantly associated with the risk of asthma development. To the best of our knowledge, this is the first investigation that demonstrated the associations of naso-pharyngeal and serum glutathione-related metabolites with the severity and asthma risk among infants hospitalized for bronchiolitis.

### 3.1. Results in Context

While research has reported that glutathione metabolism is one of the most important scavengers of reactive oxygen species [16], there is a dearth of research that examines its role in infant bronchiolitis. Within the sparse literature, an in vitro study reported that the ratio of glutathione to oxidized glutathione (glutathione disulfide) decreased in small airway epithelial cells after 24 h of RSV infection [19]. Additionally, a small single-center case–control study (n = 46) also found that the serum glutathione disulfide level was higher in infants with RSV bronchiolitis who had supplemental oxygen therapy compared to those who did not [20]. Furthermore, depletion of glutathione was also found in other respiratory diseases (e.g., cystic fibrosis [33], chronic obstructive pulmonary disease [34]). Consistently, the current study also showed a similar relationship of nasopharyngeal and serum glutathione-related metabolites with bronchiolitis severity. The relationship was similar between infants with RSV infection and those with non-RSV infection, while previous studies suggested that different viruses may invoke distinct metabolic responses [35,36,37]. This observation suggests that glutathione metabolism (and its antioxidative role) is a common pathway in the pathophysiology of bronchiolitis, regardless of the causative respiratory virus. With regard to asthma development, a small single-center case–control study (n = 30) of infants with bronchiolitis found that a higher serum serine level—a substrate that produces glycine and cysteine—was associated with the risk of asthma development [38]. Taken together, glutathione metabolism is a potential pathway that is involved in the mechanism underlying the bronchiolitis severity–incident asthma link. The current multicenter study—with a sample size many times larger than any other prior study—builds on these earlier reports and extends them by demonstrating the relationship of nasopharyngeal and serum glutathione metabolism with bronchiolitis severity and risk of asthma development.

### 3.2. Potential Mechanisms

The mechanisms underlying the observed associations of glutathione-related metabolites and bronchiolitis severity warrant clarification. First, increased oxidative stress contributes to the pathobiology of bronchiolitis [39]. Indeed, a study showed that children with bronchiolitis presented higher total oxidative status levels (based on the oxidation of ferrous ion to ferric ion) than healthy controls [17]. Additionally, an experimental study using airway epithelial cells with RSV infection also reported that higher levels of reactive oxygen species and inflammatory cytokines (e.g., IL-6, IL-8) are suppressed by intervention with synthetic catalytic scavengers in vitro [40]. Furthermore, a study using an oxidant-induced epithelial cell injury model reported that intracellular glutathione inhibits the expression of proinflammatory pathways, such as the NFκB pathway [41]. Second, respiratory virus infection may also influence the glutathione metabolism in airway epithelial cells, which antagonizes oxidative stress through converting oxidized glutathione by the action of glutathione peroxidase [42]. Indeed, an animal model study with RSV infection showed that glutathione peroxidase activity in bronchoalveolar lavage significantly decreased [39]. Taken together, these findings suggest the interplay between virus infection, oxidative stress, and glutathione metabolism, and their integrated contribution to bronchiolitis severity. Furthermore, glutathione metabolism may be a culprit in the known link between more severe bronchiolitis and the development [14,15] and pathobiology (e.g., airway hyperresponsiveness [43], airway remodeling [18]) of childhood asthma. Our data should facilitate further investigations into the underlying mechanisms that link these two common conditions.

### 3.3. Limitations

Our study has several potential limitations. First, the current study did not have non-disease controls. However, the objective was not to assess the role of the glutathione pathway in the development of bronchiolitis but to determine its relationship with bronchiolitis severity and asthma risk. Second, whereas the current study relied on the nasopharyngeal airway and serum specimens, bronchiolitis involves inflammation of the lower airway in addition to the upper airway. However, research has demonstrated that data from upper airway specimens offer a reliable representation of lung inflammation profiles [44]. Third, the current study did not directly measure the level of (reduced) glutathione, which disproportionally distributes within the cell. However, the examination of nasopharyngeal and serum glutathione-related metabolites—including glutathione substrates, downstream metabolites, and the pathway—provided a more-comprehensive view of the role of glutathione metabolism in the pathobiology of infant bronchiolitis. Fourth, the use of PPV and oxygen therapy may have altered the glutathione pathway in infants with bronchiolitis [45], while the current study did not have data on the exact timing of these therapies and specimen sampling. Fifth, as with any observational study, our causal inference may have been biased due to unmeasured confounding factors (e.g., host genetics, nutrition). Finally, even with the use of the large racially/ethnically and geographically diverse U.S. sample, the inferences must be cautiously generalized beyond infants hospitalized with bronchiolitis. Regardless, our data remain directly relevant to 110,000 hospitalized U.S. infants each year [1].

## 4. Materials and Methods

### 4.1. Study Design, Setting, and Participants

We analyzed data from a multicenter prospective observational cohort study of infants hospitalized for bronchiolitis—the MARC-35 study [15,21,23,24,25]. MARC-35 is coordinated by the Emergency Medicine Network (EMNet, www.emnet-usa.org [accessed on 4 May 2022]), an international research collaboration with 247 participating hospitals. Details of the study design, setting, participants, data collection, testing, and statistical analysis may be found in the Supplementary Materials. Briefly, MARC-35 investigators at 17 sites, across 14 U.S. states, enrolled 1016 infants (age < 1 year) who were hospitalized with an attending physician diagnosis of bronchiolitis during 3 bronchiolitis seasons (1 November to 30 April) from 2011 to 2014 (Table S1). The diagnosis of bronchiolitis was made according to the American Academy of Pediatrics bronchiolitis guidelines, defined as an acute respiratory illness with a combination of rhinitis, cough, tachypnea, wheezing, crackles, or retraction [46]. We excluded infants with a preexisting heart and lung disease, immunodeficiency, immunosuppression, or gestational age < 32 weeks. All patients were treated at the discretion of the treating physicians. The current analysis included 1013 infants enrolled in the MARC-35 study who underwent nasopharyngeal metabolome profiling. In addition, we also examined a subset of infants who underwent serum metabolome profiling, by oversampling infants with higher bronchiolitis severity (n = 140) [23]. The current analysis examines a new aim using MARC-35 cohort data and the results were not previously published. The institutional review board at each participating hospital approved the study, with written informed consent obtained from the parent or guardian.

### 4.2. Data Collection and Metabolome Profiling

Clinical data (patients’ demographic characteristics, family, environmental, and medical history, and details of the acute illness) were collected via structured interviews and chart reviews using a standardized protocol [21,23]. After the index hospitalization for bronchiolitis, we conducted parental interviews by telephone at 6-month intervals and reviewed medical records (only by trained physicians). All data were reviewed at the Emergency Medicine Network Coordinating Center at Massachusetts General Hospital (Boston, MA, USA) [47]. Investigators also collected nasopharyngeal and serum specimens within 24 h of hospitalization using standardized protocols [23]. Nasopharyngeal specimens were used for metabolomic profiling and virus testing; serum specimens were used for metabolome profiling.

The details of metabolome profiling are described in the Supplementary Materials. Briefly, the nasopharyngeal and serum metabolome profiling was conducted by Metabolon (Durham, NC, USA) using ultrahigh-performance liquid chromatography–tandem mass spectrometry. All specimens were blinded to the laboratory and processed in a random order. Instrument variability was 4%, as determined by calculating the median relative standard deviation for the internal standards.

### 4.3. Outcome Measures

The clinical outcomes of interest were acute severity of bronchiolitis and asthma at age 6 years. More specifically, the primary outcome was the use of PPV, defined as the use of continuous positive airway pressure ventilation and/or mechanical ventilation during the index hospitalization [21]. The secondary outcome was intensive care use, defined as an intensive care unit admission and/or PPV use during hospitalization [22]. Asthma was defined as a commonly used epidemiologic definition [26,27,28]: physician diagnosis of asthma, with either asthma medication use (e.g., albuterol inhaler, inhaled corticosteroids) or asthma-related symptoms (e.g., wheezing, nocturnal cough) in the preceding year.

### 4.4. Statistical Analyses

The analytic workflow is summarized in Figure 1. First, we preprocessed the metabolome data by adjusting for potential batch effect by using empirical Bayes models (ComBat method) [48]. We also normalized the nasopharyngeal metabolome data using the total sum scaling method to account for potentially differential dilutions [30] and the serum metabolome data using log-transformation [23]. We then extracted the glutathione-related metabolites data from nasopharyngeal and serum metabolome data by referring to the KEGG pathway database [29]. We computed Pearson correlation coefficients within the nasopharyngeal and serum glutathione-related metabolites to examine the correlations between these metabolites. Second, to investigate the associations of nasopharyngeal and serum glutathione-related metabolites with the outcomes of interest, we fit unadjusted and multivariable logistic regression models. In the multivariable models, we adjusted for potential confounders (age, sex, and RSV infection) that were selected based on *a priori* knowledge [35,49,50]. In the sensitivity analysis, we also stratified the analysis by RSV infection. Third, to examine whether the glutathione metabolism pathway is enriched in infants with higher bronchiolitis severity, we performed metabolite set quantitative enrichment analyses [31] using MetaboAnalyst 5.0 [51], with the Small Molecule Pathway Database library [32] as the reference. Lastly, to investigate the association of severity-related glutathione-related metabolites with asthma, we first computed glutathione-related metabolite signatures as the weighted sum of the coefficients from the generalized additive model for PPV use. Second, we examined the prediction performance of the signatures as the value of AUC. Then, we fit unadjusted and multivariable logistic regression models to examine the association of the signatures with the risk of asthma development. In the multivariable models, we adjusted for potential confounders (age, sex, race/ethnicity, prematurity, daycare use, cigarette smoke exposure at home, maternal smoking during pregnancy, parent history of eczema, RSV infection, RV infection, and immunoglobulin E sensitization) that were selected based on *a priori* knowledge [30,49,50,52,53]. Statistical analyses were conducted using R version 4.1.0 (R Foundation, Vienna, Austria). All *p*-values were two-tailed, with *p* < 0.05 considered statistically significant. We accounted for multiple testing using the Benjamini–Hochberg FDR method [54].

## 5. Conclusions

Based on the multicenter analysis of large nasopharyngeal and serum metabolome data from infants hospitalized for bronchiolitis, we identified glutathione-related metabolites that were associated with bronchiolitis severity. Furthermore, the nasopharyngeal metabolite signature for higher severity was associated with the risk of asthma development. For clinicians, our findings suggest the potential role of glutathione-related metabolites in the identification of infants at risk for higher severity and asthma development. For researchers, our data advance research not only into the discovery of drug targets for bronchiolitis [55] but also into the mechanism underlying the bronchiolitis severity–incident asthma link.

## Figures and Tables

**Table 1:** Characteristics and clinical course of infants hospitalized for bronchiolitis, according to nasopharyngeal and serum metabolome measurement.

Patient Characteristics	Nasopharyngeal Sample (n = 1013)	Serum Sample (n = 140)
**Demographics**		
Age (month), median (IQR)	3 (2–6)	3 (1–6)
Female sex	406 (40)	53 (38)
Race/ethnicity		
Non-Hispanic white	428 (42)	54 (39)
Non-Hispanic black	239 (24)	29 (21)
Hispanic	308 (30)	53 (38)
Other or unknown	38 (4)	4 (3)
C-section delivery	347 (35)	52 (37)
Prematurity (32–36.9 weeks)	186 (18)	34 (24)
History of eczema	149 (15)	20 (14)
Ever attended daycare	233 (23)	24 (17)
Cigarette smoke exposure at home	155 (15)	15 (11)
Maternal smoking during pregnancy	147 (15)	17 (12)
Parent history of eczema	198 (20)	32 (23)
Previous breathing problems (count)		
0	808 (80)	106 (76)
1	159 (16)	24 (17)
2	46 (5)	10 (7)
Previous ICU admission	17 (2)	5 (4)
**Clinical presentation at index hospitalization**		
Weight (kg), median (IQR)	6.1 (4.7–7.7)	6.0 (4.4–7.8)
Respiratory rate (per min), median (IQR)	48 (40–60)	48 (40–60)
Oxygen saturation at presentation		
<90%	91 (9)	17 (12)
90–93%	155 (16)	26 (19)
≥94%	746 (75)	94 (69)
Respiratory virus		
RSV	818 (81)	97 (69)
Rhinovirus	213 (21)	55 (39)
Other pathogens *	237 (23)	37 (26)
Laboratory data		
Any IgE sensitization ^†^	203 (20)	28 (20)
Clinical outcomes		
Positive pressure ventilation use ^‡^	55 (5)	38 (27)
Intensive care use ^§^	163 (16)	70 (50)
Length of hospital stay (days), median (IQR)	2 (1–3)	3 (2–6)
Asthma at age 6 years ^||^	328 (32)	68 (49)

Abbreviations: IgE, immunoglobulin E; IQR, interquartile range; RSV, respiratory syncytial virus. Data are n (%) of infants, unless otherwise indicated. Percentages may not equal 100 because of rounding and missingness. * Adenovirus, bocavirus, Bordetella pertussis, enterovirus, human coronavirus NL63, OC43, 229E, or HKU1, human metapneumovirus, influenza A or B virus, Mycoplasma pneumoniae, and parainfluenza virus 1–3. † Defined by having one or more positive values for allergen-specific IgE at index hospitalization. ‡ Defined as the use of continuous positive airway pressure ventilation and/or mechanical ventilation during the hospitalization. § Defined as admission to the intensive care unit and/or the use of continuous positive airway pressure ventilation and/or mechanical ventilation during hospitalization. || Defined by physician diagnosis of asthma by age six years, plus either asthma medication use (e.g., albuterol inhaler, inhaled corticosteroids, montelukast) or asthma-related symptoms in the preceding year.

## Data Availability

The data presented in this study are available upon reasonable request from the researchers, whose research investigates severe bronchiolitis, recurrent wheezing, asthma, and related concepts. The data are not publicly available to be compliant with the informed consent forms of the MARC-35 study and the data sharing plan.

## Supplementary Materials

The following supporting information can be downloaded at: https://www.mdpi.com/article/10.3390/metabo12080674/s1, Supplementary methods; Table S1: Principal investigators at the 17 participating sites in MARC-35; Figure S1: Pathway of glutathione-related metabolites; Figure S2: Correlations between serum glutathione-related metabolites and associations of glutathione-related metabolites with severity outcomes in infants hospitalized for bronchiolitis; Figure S3: Prediction ability of glutathione-related metabolite signatures for positive pressure ventilation use in infants hospitalized for bronchiolitis. References [56,57,58,59,60] are cited in Supplementary Materials.
