# Supplementary material for: Association of Nasopharyngeal and Serum Glutathione Metabolism with Bronchiolitis Severity and Asthma Risk: A Prospective Multicenter Cohort Study
Source: Metabolites. 2022 Jul 22;12(8):674. doi: 10.3390/metabo12080674 (PMC9394245; doi:10.3390/metabo12080674)
Supplement: Supplementary file 1 [file metabolites-12-00674-s001.zip › metabolites-1806813-supplementary.pdf]

Supplementary Materials

Association of Nasopharyngeal and Serum Glutathione Metabolism with Bronchiolitis Severity and Asthma Risk: A Prospective Multicenter Cohort Study

Table of Contents

Supplementary Methods

Supplementary References

Table S1. Principal investigators at the 17 participating sites in MARC-35

Figure S1. Pathway of glutathione-related metabolites

Figure S2. Correlations between serum glutathione-related metabolites and associations of glutathione-related metabolites with severity outcomes in infants hospitalized for bronchiolitis

Figure S3. Prediction ability of glutathione-related metabolite signatures for positive pressure ventilation use in infants hospitalized for bronchiolitis

SUPPLEMENTARY METHODS

Study design, setting, and participants

This is an analysis of data from the 35th Multicenter Airway Research Collaboration (MARC-35)—a multicenter prospective cohort study of infants hospitalized with bronchiolitis (i.e., severe bronchiolitis). [22] MARC-35 is coordinated by the Emergency Medicine Network (EMNet), a collaboration of 247 participating hospitals. [56] Site investigators enrolled infants (age <1 year) hospitalized with bronchiolitis at 17 sites across 14 U.S. states (**Table S1**) using a standardized protocol during three consecutive bronchiolitis seasons (from November 1 through April 30) during 2011-2014. Bronchiolitis was defined by the American Academy of Pediatrics guidelines as an acute respiratory illness with some combination of rhinitis, cough, tachypnoea, wheezing, crackles, and retractions [46]; and was diagnosed by an attending physician. We excluded infants who were transferred to a participating hospital >24 hours after the original hospitalization, those who were consented >24 hours after hospitalization, or those with a known heart-lung disease, immunodeficiency, immunosuppression, or gestational age of <32 weeks. All patients were treated at the discretion of the treating physicians. The institutional review board at each of the participating hospitals approved the study. Written informed consent was obtained from the parent or guardian.

Data collection

Clinical data (patients' demographic characteristics, and family, environmental, and medical history, and details of the acute illness) were collected via structured interview and chart reviews. [22] All data were reviewed at the EMNet Coordinating Center (Boston, MA), and site investigators were queried about missing data and discrepancies identified by manual data checks. In addition to the clinical data, nasopharyngeal airway and serum samples were collected by trained site investigators using the standardized protocol that was utilized in a previous cohort study of children with severe bronchiolitis. [52] All sites used the same collection equipment (Medline Industries, Mundelein, IL, USA) and collected the samples within 24 hours of hospitalization. For the collection, the infant was placed supine, 1 mL of normal saline was instilled into one naris, and then an 8 French

suction catheter was used to remove the mucus. This procedure was performed once on each nostril. After the sample collection from both nares, 2 mL of normal saline was suctioned through the catheter to clear the tubing and to ensure that a standard volume of aspirate is obtained. The nasopharyngeal airway and serum specimens were immediately placed on ice and then stored at −80°C. Frozen samples were shipped in batches to 1) Metabolon (Durham, North Carolina, USA) for metabolomic profiling and 2) Baylor College of Medicine (Houston, Texas, USA) for respiratory viruses using real-time polymerase chain reaction (RT-PCR).

Nasopharyngeal airway and serum metabolomic profiling

Metabolomic profiling used 125 μl of each nasopharyngeal airway sample and 100 μl of each serum sample. All samples were blinded to Metabolon and processed in a random order. Sample preparation was carried out as described previously. [57] Recovery standards were added prior to the first step in the extraction process for quality control purposes. Proteins were precipitated with methanol under vigorous shaking for 2 minutes (Glen Mills Genogrinder 2000; Clifton, NJ) followed by centrifugation. The resulting extract was divided into four fractions: two (i.e., early and late eluting compounds) for analysis by ultra-high performance liquid chromatography-tandem mass spectrometry UPLC-MS/MS (positive ionization), one for analysis by UPLC-MS/MS (negative ionization), and one for the UPLC-MS/MS polar platform (negative ionization). Three types of controls were analyzed in concert with the experimental samples: samples generated from a pool from a small portion of each experimental sample of interest served as technical replicate throughout the data set; extracted water samples served as process blanks; a cocktail of standards spiked into every analyzed sample allowed instrument performance monitoring. The median relative standard deviation for the standards that were added to each sample—a measure of instrument variability—was <5%.

Ultrahigh Performance Liquid Chromatography-Tandem Mass Spectroscopy (UPLC-MS/MS)

All methods utilized a Waters ACQUITY UPLC (Milford, MA) and a Thermo Scientific Q-Exactive (Waltham, MA) high resolution/accurate MS interfaced with a heated electrospray ionization (HESI-II) source and Orbitrap mass analyzer operated at 35,000 mass resolution. The sample extract was dried and then reconstituted in solvents compatible with each of the four methods. Each reconstitution solvent contained a series of standards at fixed concentrations to ensure injection and chromatographic consistency. One aliquot was analyzed using acidic positive ion conditions, chromatographically optimized for more hydrophilic compounds. In this method, the extract was gradient eluted from a C18 column (Waters UPLC BEH C18-2.1x100 mm, 1.7 μm) using water and methanol, containing 0.05% perfluoropentanoic acid and 0.1% formic acid. Another aliquot was also analyzed using acidic positive ion conditions, but it was chromatographically optimized for more hydrophobic compounds. In this method, the extract was gradient eluted from the same aforementioned C18 column using methanol, acetonitrile, water, 0.05% perfluoropentanoic acid, and 0.01% formic acid and was operated at an overall higher organic content. Another aliquot was analyzed using basic negative ion optimized conditions using a separate dedicated C18 column. The basic extracts were gradient eluted from the column using methanol and water, including 6.5mM Ammonium Bicarbonate at pH 8. The fourth aliquot was analyzed via negative ionization following elution from a HILIC column (Waters UPLC BEH Amide 2.1x150 mm, 1.7 μm) using a gradient consisting of water and acetonitrile with 10mM Ammonium Formate, pH 10.8. The MS analysis alternated between MS and data-dependent MS^n scans using dynamic exclusion. The scan range varied slightly between methods but covered 70-1000 m/z. Metabolites were identified by automated comparison of the ion features in the experimental samples to a reference library of chemical standard entries that include retention time, molecular weight (m/z), preferred adducts, and in-source fragments as well as associated MS spectra, and curated by visual inspection for quality control using QUICS software. [58] Identification of known chemical entities was based on comparisons to metabolomic library entries of >3,000

purified standards. Peaks were quantified using area-under-the-curve. Raw area counts for each metabolite in each sample were normalized to correct for variation due to instrument inter-day tuning differences by the median value for each run-day. Missing values were imputed with the observed minimum for that particular compound.

Four types of quality controls were analyzed in concert with the specimens: 1) samples generated from a pool from a small portion of each experimental specimen that served as technical replicate; 2) extracted water samples that served as process blanks; 3) samples of solvent used in extraction; and 4) a cocktail of standards spiked into every analyzed specimen that allowed instrument performance monitoring. The median relative standard deviation (RSD) for the standards that are added to each sample—a measure of instrument variability—was <5%.

Respiratory virus measurement

We identified respiratory viruses using singleplex or duplex two-step RT-PCR at Baylor College of Medicine (Houston, TX, USA). We used real-time reverse transcriptase-PCR to detect RNA respiratory viruses, including respiratory syncytial virus (RSV) [60] and 13 other RNA viruses. All RT-PCR assays were tested in duplicate and samples with incongruent values (one well positive) were retested. To reduce carryover contamination, sample preparation, RNA/DNA extraction, cDNA, and amplification were performed in separate areas. All PCR runs had extraction and reagent positive and negative controls.

SUPPLEMENTARY REFERENCES

22. Hasegawa, K.; Mansbach, J.M.; Ajami, N.J.; Espinola, J.A.; Henke, D.M.; Petrosino, J.F.; Piedra, P.A.; Shaw, C.A.; Sullivan, A.F.; Camargo, C.A.; the MARC-35 Investigators. Association of nasopharyngeal microbiota profiles with bronchiolitis severity in infants hospitalised for bronchiolitis. *Eur. Respir. J.* **2016**, *48*, 1329–1339. https://doi.org/10.1183/13993003.00152-2016.

56. Emergency Medicine Network. Available online: http://www.emnet-usa.org/ (accessed on 4 May 2022).

46. Ralston, S.L.; Lieberthal, A.S.; Meissner, H.C.; Alverson, B.K.; Baley, J.E.; Gadomski, A.M.; Johnson, D.W.; Light, M.J.; Maraqa, N.F.; Mendonca, E.A.; et al. Clinical practice guideline: The diagnosis, management, and prevention of bronchiolitis. *Pediatrics* **2014**, *134*, e1474–e1502. https://doi.org/10.1542/peds.2014-2742.

52. Hasegawa, K.; Jartti, T.; Mansbach, J.M.; Laham, F.R.; Jewell, A.M.; Espinola, J.A.; Piedra, P.A.; Camargo, C.A. Respiratory syncytial virus genomic load and disease severity among children hospitalized with bronchiolitis: Multicenter cohort studies in the United States and Finland. *J. Infect. Dis.* **2015**, *211*, 1550–1559. https://doi.org/10.1093/infdis/jiu658.

57. Evans, A. M.; DeHaven, C. D.; Barrett, T.; Mitchell, M.; Milgram, E. Integrated, nontargeted ultrahigh performance liquid chromatography/electrospray ionization tandem mass spectrometry platform for the identification and relative quantification of the small-molecule complement of biological systems. *Anal Chem* **2009**, *81*, 6656-6667. https://doi.org/10.1021/ac901536h.

58. Dehaven, C. D.; Evans, A. M.; Dai, H.; Lawton, K. A. Organization of GC/MS and LC/MS metabolomics data into chemical libraries. *J Cheminform* **2010**, *2*, 9. https://doi.org/10.1186/1758-2946-2-9.

59. van Elden, L. J. R.; van Loon, A. M.; van der Beek, A.; Hendriksen, K. A. W.; Hoepelman, A. I. M.; van Kraaij, M. G. J.; Schipper, P.; Nijhuis, M. Applicability of a real-time quantitative PCR assay for diagnosis of respiratory syncytial virus infection in immunocompromised adults. *J Clin Microbiol* **2003**, *41*, 4378-4381. https://doi.org/10.1128/JCM.41.9.4378-4381.2003.

60. Kuypers, J.; Wright, N.; Ferrenberg, J.; Huang, M.-L.; Cent, A.; Corey, L.; Morrow, R. Comparison of real-time PCR assays with fluorescent-antibody assays for diagnosis of respiratory virus infections in children. *J Clin Microbiol* **2006**, *44*, 2382-2388. https://doi.org/10.1128/JCM.00216-06.

Supplementary Table S1. Principal investigators at the 17 participating sites in MARC-35.

**Amy D. Thompson, MD**	**Alfred I. duPont Hospital for Children, Wilmington, DE**
Federico R. Laham, MD, MS	Arnold Palmer Hospital for Children, Orlando, FL
Jonathan M. Mansbach, MD, MPH	Boston Children's Hospital, Boston, MA
Vincent J. Wang, MD, MHA and Susan Wu, MD	Children's Hospital of Los Angeles, Los Angeles, CA
Michelle B. Dunn, MD and Jonathan M. Spergel, MD, PhD	Children's Hospital of Philadelphia, Philadelphia, PA
Juan C. Celedón, MD, DrPH	Children's Hospital of Pittsburgh, Pittsburgh, PA
Michael R. Gomez, MD, MS-HCA and Nancy Inhofe, MD	The Children's Hospital at St. Francis, Tulsa, OK
Brian M. Pate, MD and Henry T. Puls, MD	The Children's Mercy Hospital & Clinics, Kansas City, MO
Stephen J. Teach, MD, MPH	Children's National Medical Center, Washington, D.C.
Richard T. Strait, MD and Stephen C. Porter, MD, MSc, MPH	Cincinnati Children's Hospital and Medical Center, Cincinnati, OH
Ilana Y. Waynik, MD	Connecticut Children's Medical Center, Hartford, CT
Sujit Iyer, MD	Dell Children's Medical Center of Central Texas, Austin, TX
Michelle D. Stevenson, MD, MS	Norton Children's Hospital, Louisville, KY
Wayne G. Shreffler, MD, PhD and Ari R. Cohen, MD	Massachusetts General Hospital, Boston, MA
Anne K. Beasley, MD and Cindy S. Bauer, MD	Phoenix Children's Hospital, Phoenix, AZ
Thida Ong, MD and Markus Boos, MD, PhD	Seattle Children's Hospital, Seattle, WA
Charles G. Macias, MD, MPH	Texas Children's Hospital, Houston, TX

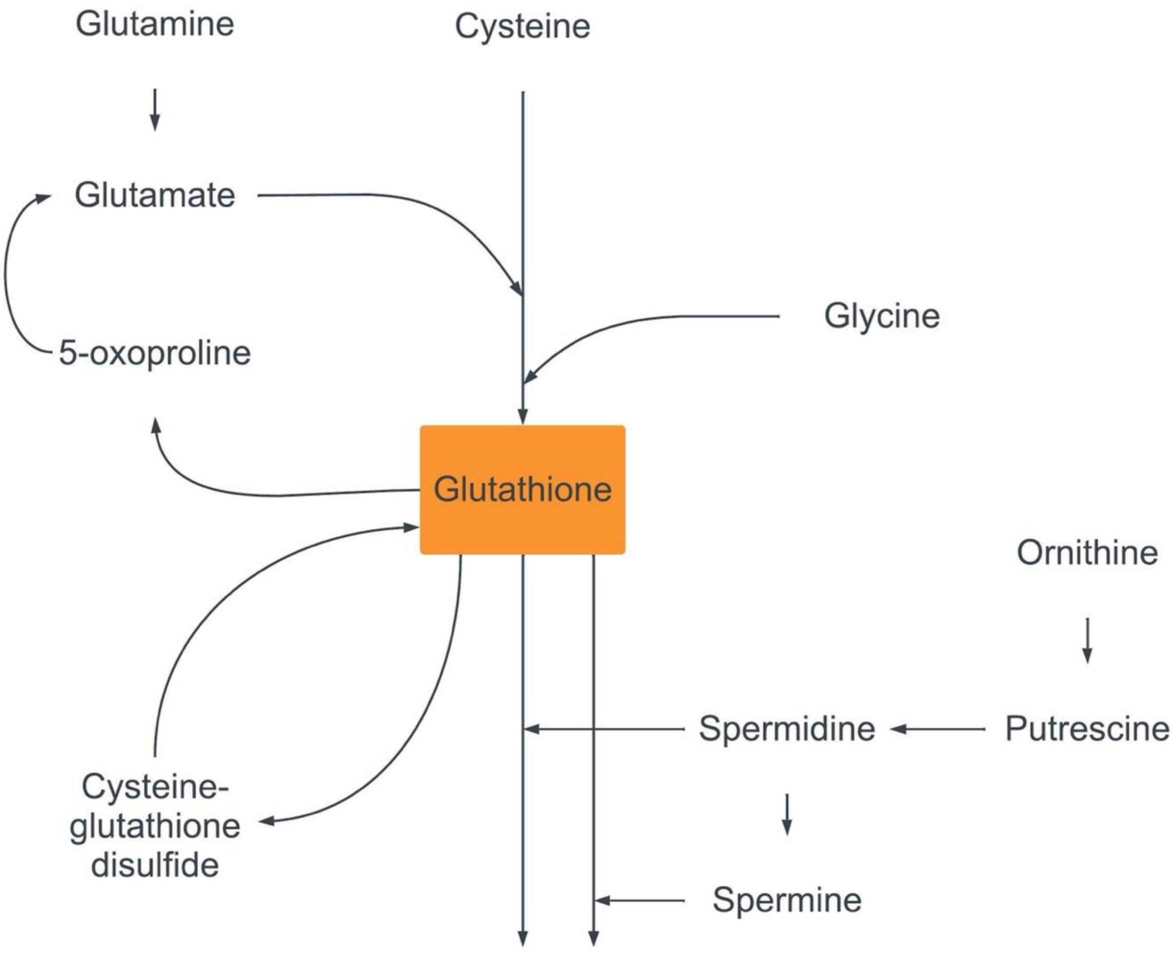

Supplementary Figure S1. Pathway of glutathione-related metabolites. The pathway of glutathione-related metabolites is shown referring to the KEGG pathway database. Abbreviation: KEGG, Kyoto Encyclopedia of Genes and Genomes.

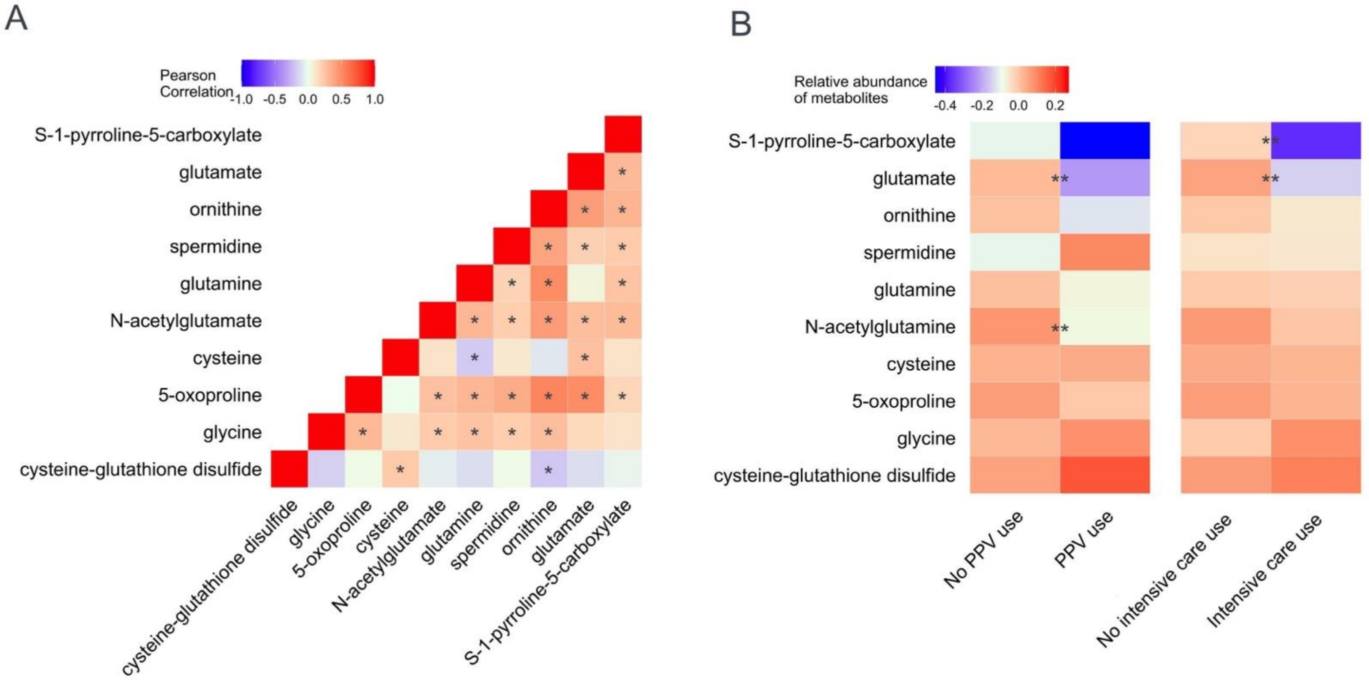

Supplementary Figure S2. Correlations between serum glutathione-related metabolites and associations of glutathione-related metabolites with severity outcomes in infants hospitalized for bronchiolitis. A. The heatmap shows the correlation between 10 measured serum glutathione-related metabolites. Glutathione substrates (e.g.,-cysteine and glutamate) were positively correlated with each other. * P-value <0.05 estimated by Pearson correlation coefficient.. B. The heatmap shows the association of serum glutathione-related metabolites with each of the two clinical outcomes. The relative abundance of glutamate (a substrate of glutathione) was non-significantly lower in the PPV use group and the intensive care use group compared to their reference groups, suggesting their depletion. The association of each metabolite with each outcome is shown in Figure 3. ** FDR <0.10 estimated by unadjusted logistic regression models. Abbreviations: FDR, false discovery rate; PPV, positive pressure ventilation.

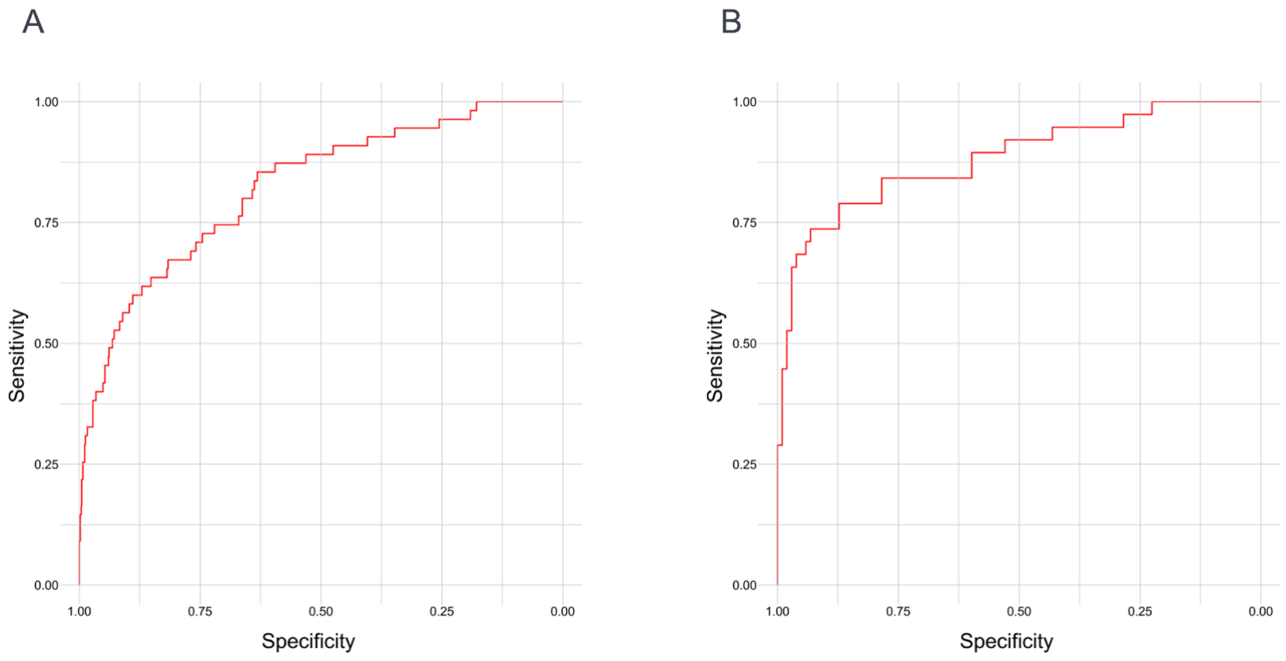

Supplementary Figure S3. Prediction ability of glutathione-related metabolite signatures for positive pressure ventilation use in infants hospitalized for bronchiolitis. A. Nasopharyngeal glutathione-related metabolite signature: The ROC curve shows the prediction ability of the signature for PPV use, with an AUC of 0.82 (95%CI 0.76-0.88). B. Serum glutathione-related metabolite signature: The ROC curve shows the prediction ability of the signature for PPV use, with an AUC of 0.88 (95%CI 0.81-0.95). Abbreviations: AUC, area under the receiver-operating-characteristics curve; CI, confidence interval; PPV, positive pressure ventilation; ROC, receiver-operating-characteristics.

SUPPLEMENTARY REFERENCES

22. Hasegawa, K.; Mansbach, J.M.; Ajami, N.J.; Espinola, J.A.; Henke, D.M.; Petrosino, J.F.; Piedra, P.A.; Shaw, C.A.; Sullivan, A.F.; Camargo, C.A.; the MARC-35 Investigators. Association of nasopharyngeal microbiota profiles with bronchiolitis severity in infants hospitalised for bronchiolitis. *Eur. Respir. J.* **2016**, *48*, 1329–1339. https://doi.org/10.1183/13993003.00152-2016.

56. Emergency Medicine Network. Available online: http://www.emnet-usa.org/ (accessed on 4 May 2022).

46. Ralston, S.L.; Lieberthal, A.S.; Meissner, H.C.; Alverson, B.K.; Baley, J.E.; Gadomski, A.M.; Johnson, D.W.; Light, M.J.; Maraqa, N.F.; Mendonca, E.A.; et al. Clinical practice guideline: The diagnosis, management, and prevention of bronchiolitis. *Pediatrics* **2014**, *134*, e1474–e1502. https://doi.org/10.1542/peds.2014-2742.

52. Hasegawa, K.; Jartti, T.; Mansbach, J.M.; Laham, F.R.; Jewell, A.M.; Espinola, J.A.; Piedra, P.A.; Camargo, C.A. Respiratory syncytial virus genomic load and disease severity among children hospitalized with bronchiolitis: Multicenter cohort studies in the United States and Finland. *J. Infect. Dis.* **2015**, *211*, 1550–1559. https://doi.org/10.1093/infdis/jiu658.

57. Evans, A. M.; DeHaven, C. D.; Barrett, T.; Mitchell, M.; Milgram, E. Integrated, nontargeted ultrahigh performance liquid chromatography/electrospray ionization tandem mass spectrometry platform for the identification and relative quantification of the small-molecule complement of biological systems. *Anal Chem* **2009**, *81*, 6656-6667. https://doi.org/10.1021/ac901536h.

58. Dehaven, C. D.; Evans, A. M.; Dai, H.; Lawton, K. A. Organization of GC/MS and LC/MS metabolomics data into chemical libraries. *J Cheminform* **2010**, *2*, 9. https://doi.org/10.1186/1758-2946-2-9.

59. van Elden, L. J. R.; van Loon, A. M.; van der Beek, A.; Hendriksen, K. A. W.; Hoepelman, A. I. M.; van Kraaij, M. G. J.; Schipper, P.; Nijhuis, M. Applicability of a real-time quantitative PCR assay for diagnosis of respiratory syncytial virus infection in immunocompromised adults. *J Clin Microbiol* **2003**, *41*, 4378-4381. https://doi.org/10.1128/JCM.41.9.4378-4381.2003.

60. Kuypers, J.; Wright, N.; Ferrenberg, J.; Huang, M.-L.; Cent, A.; Corey, L.; Morrow, R. Comparison of real-time PCR assays with fluorescent-antibody assays for diagnosis of respiratory virus infections in children. *J Clin Microbiol* **2006**, *44*, 2382-2388. https://doi.org/10.1128/JCM.00216-06.
